# The Control and Prevention of COVID-19 Transmission in Children: A Protocol for Systematic Review and Meta-analysis

**DOI:** 10.1097/MD.0000000000021393

**Published:** 2020-07-31

**Authors:** Gidyenne Christine Bandeira Silva de Medeiros, Ana Clara de França Nunes, Kesley Pablo Morais de Azevedo, Victor Hugo de Oliveira Segundo, Gilberto Martins Santos, Ádala Nayana de Sousa Mata, Isac Davidson Pimenta, Isaac Newton Machado Bezerra, Liliane Pereira Braga, Helaine Carneiro Capucho, Márcia Regina Piuvezam, Valter Cordeiro Barbosa Filho, José Carlos Leitão, Daniel Guillén Martínez, Grasiela Piuvezam

**Affiliations:** a Department of Public Health, Postgraduate Program in Public Health, Federal University of Rio Grande do Norte, Natal/RN, Brazil; b Department of Administration (Campus PM), Postgraduate Program in Management of Public Organizations, Federal University of Santa Maria, Santa Maria/RS, Brazil; c Multicampi School of Medical Sciences of RN, Federal University of Rio Grande do Norte, Caicó/RN, Brazil; d Multiprofessional Residency Program of Interiorization Health Care, Federal University of Pernambuco, Vitória de Santo Antão/PE, Brazil; e Department of Pharmacy, Faculty of Health Sciences, University of Brasilia (UnB); f Center for Health Sciences, Federal University of Paraíba (UFPB), Paraiba, Brazil; g Federal Institute of Ceará — Aracati Campus, Brazil; h Research Center in Sports Sciences, Health and Human Development, CIDESD, Vila Real, Portugal; i Faculty of Nursing, Catholic University of Murcia (UCAM), Murcia, Spain.

**Keywords:** children, adolescents, COVID-19, SARS-CoV-2 infection

## Abstract

**Background:**

The pandemic following the rapid spread of the new SARS-CoV-2 virus has hit all continents and caused thousands of deaths worldwide. Evidence has been published on epidemiological and clinical characteristics of population groups considered at risk; however, information for the other population groups, especially for the child population, is needed. In this context, this protocol describes a systematic review that will aim to identify the evidence on control and prevention of COVID-19 transmission among children and adolescents, as well as to describe the epidemiological profile and clinical and immunological characteristics of COVID-19 in this population.

**Methods:**

This protocol will be developed in accordance with PRISMA-P. The searches will be conducted in PubMed, Web of Science, ScienceDirect, EMBASE, and Scopus, seeking clinical trials. Observational studies and case reports with Children and adolescents (≤19 years) infected with SARS-CoV-2 will be included whether they report information on the control of prevention and COVID-19 transmission. Two independent researchers will perform the selection of articles, removal of duplication, and screening by Rayyan QCRI application. Cochrane's RoB 2.0, ROBINS-I, and CASP tools will be used to assess the risk of bias. Meta-analysis, subgroup analyses, and/or descriptive analyses will be carried out based on the data conditions included.

**Results:**

A high-quality synthesis of the available evidences on the epidemiological profile, the clinical and immunological characteristics involved in children, and adolescents diagnosed with COVID-19, as well as the participation of this population in the transmission dynamics of SARS-CoV-2 will be provided.

**Conclusion:**

This systematic review has an important relevance in the current context because it has a great potential to help the development of new control and prevention strategies in the pediatric population.

**Record of systematic review:**

CRD42020179263.

## 1 Introduction

The contemporary pandemic declared by the World Health Organization (WHO) after the rapid spread of the new Severe Acute Respiratory Syndrome-Coronavirus 2 (SARS-CoV-2 virus) has hit all continents and caused thousands of deaths worldwide. WHO later named it a disease caused by coronavirus 2019 or COVID-19.^[1]^

The observed and published data on the infection and mortality rates of COVID-19 point to the existence of more vulnerable groups, especially elderly individuals and patients with chronic diseases. In this sense, many information has been published on the epidemiological and clinical characteristics of these population groups.^[2]^ However, technical and scientific information are needed for the other population groups, especially for the infant population.

A recent systematic review study analyzed publications on COVID-19 in the infant population and found a reduced possibility of contagion (dissemination) in this group ranging from 1% to 5%.^[3]^ The explanatory hypothesis that stands out is the insufficient rate of tests performed in children, mainly because most of these individuals are asymptomatic, according to a study by Bi et al (2020) that analyzed >2000 children with COVID-19 and >90% of them were asymptomatic or had very mild symptoms.^[4]^

Another systematic review study (Castagnoli et al, 2020) that approached the main clinical features and management of pediatric cases of SARS-CoV-2 infection showed that most of the evidence results from clinical studies and cases in China. The summary of the evidence indicated that children mainly acquire SARS-CoV-2 infection from their relatives, but seem to experience a less severe form of the disease compared to adults, presenting mild symptoms and in cases of good prognosis, there is recovery within 1 to 2 weeks.^[5]^ These findings are corroborated by the study by Rasmissen and Thompson 2020; however, the authors also highlight that there are gaps, especially with regard to the effects of the disease in children with special health needs.^[6]^ Another research conducted with Spanish children also indicated the need for additional information to trace the characteristics of the disease in this population.^[7]^

In this context, considering that this infant population resides with other individuals (parents, grandparents, or guardians) the probability of becoming transmitters of COVID-19 to family members becomes high, especially because they are asymptomatic. Thus, in order for new control and prevention strategies to be idealized, understanding the dynamics of transmission and control of the virus in this population becomes relevant, which refers to the following questioning: what evidence is available in the literature that addresses the participation of children and adolescents, as asymptomatic carriers, in the control and prevention of COVID-19 transmission?

This protocol describes the method of a systematic review that will aim to identify the evidence available in the literature on the participation of the child population in the control and prevention of COVID-19 transmission, and also describe the epidemiological profile and clinical and immunological characteristics of COVID-19 in this population. A secondary aim of the review will be to map the gradient of contribution of this population in the dynamics of disease transmission.

## 2 Methods and analysis

### 2.1 Protocol and registration

This systematic review was recorded in the International prospective register of Systematic reviews (PROSPERO) on May 4, 2020 under the number CRD42020179263. Available at: https://www.crd.york.ac.uk/prospero/display_record.php?ID=CRD42020179263.

### 2.2 Analysis Plan

**Figure 1:**
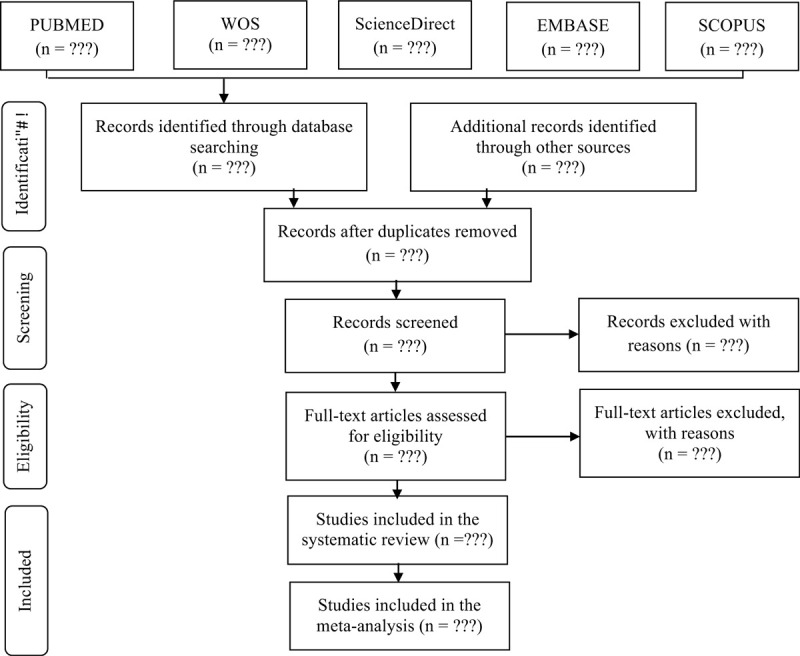
Flow diagram. Adapted from PRISMA-P.

This protocol will be developed in accordance with the Preferred Reporting Items for Systematic Reviews and Meta-Analyses Protocols (PRISMA-P),^[8,9]^ and systematic review according to the Preferred Reporting Items for Systematic Reviews and Meta-Analyses (PRISMA),^[10]^ which provides a selection flow diagram (Fig. 1).

### 2.3 Search strategy

The searches will be conducted in 5 electronic databases (PubMed, Web of Science, ScienceDirect, EMBASE, and Scopus) by 2 independent researchers (ACFN and VHOS), who will apply the search strategies using the following terms: children, Child, Adolescent, Student, Young children, Young people, Pupils, School age; COVID-19, SARS-CoV-2 infection, Coronavirus disease-19; transmission; Prevention and control, with boolean operators AND and OR used between the terms. The Rayyan QCRI^[11]^ application (specific for systematic reviews) will be used to read titles and abstracts, remove duplicates, and read full texts. And for the management of references will be used the Software Mendeley.^[12]^ Initially, the reading by title and abstract will be performed, after the removal of duplicates, and then, the complete reading of the selected articles. If any abstract is not provided using the search strategy, the full text of the manuscript will be reviewed and evaluated. If the 2 independent researchers disagree about the inclusion of any study in the review, a third researcher (GP) will decide whether or not to include the study.

### 2.4 Eligibility Criteria

#### 2.4.1 Types of studies

Clinical trials, observational studies, and case reports describing the epidemiological profiles and clinical-immunological characteristics of COVID-19 in children and adolescents confirmed with SARS-CoV-2 infection, according to WHO guidelines; and the participation of the child population in the transmission dynamics of COVID-19.

#### 2.4.2 Population

Children and adolescents (≤19 years)^[13]^ infected with SARS-CoV-2 were confirmed by the RT-PCR or serology method to identify previous infection and WHO diagnostic criteria.

#### 2.4.3 Intervention

For the intervention effect, SARS-CoV-2 infection will be considered, as well as the control and prevention of COVID-19 transmission in children and adolescents.

#### 2.4.4 Comparison

Several control interventions will be included: level of severity of infection (severe/intensive care units (ICU) versus nonsevere/non-ICU); existence of prespecified comorbidities (diabetes mellitus, hypertension, chronic obstructive pulmonary disease, cardiovascular diseases, and others) versus no comorbidities; children and adolescents with COVID-19 who died will be compared with those who did not die; by geographic region.

#### 2.4.5 Exclusion criteria

Studies addressing children and adolescents populations with psychological or intellectual disabilities. Studies including only children and adolescents with psychological or intellectual disabilities (mental retardation).

#### 2.4.6 Types of outcomes

The main expected results include data on the mean proportion or 95% confidence interval of: incidence and mortality due to COVID-19 disease; early comorbidities (diabetes, hypertension, and); complications (acute respiratory distress syndrome, acute kidney injury, and so on); the results of the course of the disease (hospitalization, discharge, deaths, and so on); descriptions and data on the participation of the child population in the transmission dynamics of COVID-19; clinical and immunological characteristics of COVID-19 in the infant population; demographic data (age, sex, race, country, and so on) and clinical symptoms (fever, cough, and so on).

#### 2.4.7 Data synthesis and analysis

The extraction of all data will be done in a standardized manner by 2 independent authors (KPMA and IDP), creating a database in a predesigned spreadsheet and tested earlier in the Excel program. The following information will be presented in this database: identification of the studies (first author, author's name, year of publication, period of study, geographic region); participant's data (age, sample size, sex, period and duration of recruitment); epidemiological information; clinical characteristics; data on the participation of children and adolescents in the transmission dynamics of COVID-19. For any relevant data lost, we will contact the authors of the study. If we do not receive the necessary information, or in the face of the impossibility of imputation of the data, the results will be deleted from our analysis and will be covered in the Discussion section in the discussion section, only qualitatively.

If there is a methodological homogeneity in the studies included (at least 2) in the systematic review, a meta-analysis will be performed. The evaluation of heterogeneity between the studies will be performed by the statistical tests *χ*^2^ and *I*^2^. Random-effect or fixed-effect models will then be applied to calculate the total size of the effect of the studies included in the meta-analysis. If, however, the studies are considered too heterogeneous, only a narrative synthesis will be performed. The following criteria will be used: 0% to 40%, might not be important; 30% to 60%, may represent moderate heterogeneity; 50% to 90%, may represent substantial heterogeneity; 75% to 100%, considerable heterogeneity.^[14]^ For case studies included with qualitative evidence, a metasynthesis approach will be adopted.

### 2.5 Subgroup analysis

Subgroup analyses will be performed if relevant data are available. If possible, the following will be performed: an analysis of subgroups based on the children's ages (newborns [0–4 weeks]; infants [4 weeks–1 year]; children [12–24 months]; preschoolers [2–5 years; school-age children [6–12 years]; and adolescents [13–19 years]); subgroup analysis based on location, comorbidities mentioned previously, and laboratory characteristics. In addition, the data will be analyzed according to the drawings of the studies.

### 2.6 Bias risk assessment

The risk of bias assessment will be performed in a standardized manner by 2 independent authors (KPMA and GCBSM), and disagreements will be defined during consensus meetings. To assess the risk of bias in the experimental studies included, the Cochrane risk of bias tool (RoB 2.0)^[15]^ will be applied, whereas nonrandomized studies will be evaluated using a Risk of bias tool to assess nonrandomized studies of interventions (ROBINS-I).^[16]^

If qualitative studies are included, the risk of bias will be assessed using the Critical Appraisal Skills Program verification checklist,^[17]^ recommended by the Cochrane Collaboration for qualitative literature.

### 2.7 Ethics and dissemination

Ethical approval and informed consent are not necessary for this research, because it is a systematic review (use of secondary data).

## 3 Discussion

At the initial stage of the outbreak, COVID-19 was more prevalent in people aged 15 years or older, and the proportion of confirmed cases was relatively small in children. Since then, no special prevention and control measures have been adopted in this population, and the number of cases of childhood infection has increased significantly, especially in younger age groups.^[18]^ Associated with this, children exhibit certain particularities and cannot clearly describe their own health status or contact history, which contributed to the serious challenge of protecting, diagnosing, and treating this population.^[19]^

Although children and young adults are clearly susceptible to SARS-CoV-2 infection, attention has focused primarily on their potential role in influencing dissemination and transmission in the community. Data reanalyzed from the epicenter of the Chinese outbreak observed that children accounted for 12% of infections.^[20]^ However, understanding the protection mechanism in children can help in the development of therapeutic goals for the population considered primarily at risk, such as the elderly. In addition, it is also important to identify the risks in children to make recommendations on therapy and, where available, vaccines.^[21]^

In the study conducted by DeBiasi et al (2020), from their cohort of 177 pediatric patients, the authors highlighted the potential for the worsening of the disease in this age group, and sent an important warning to that other regions could adopt measures of anticipation (prevention) and response (therapy) against COVID-19, including a significant burden of children and young adults hospitalized and in critical condition.^[22]^

We hope that the completed systematic review helps to understand the main clinical features and management of pediatric cases of SARS-CoV-2 infection and the dynamics of transmission and control of the virus in this population. Moreover, it may also help to define which strategies should be used to control and prevention of COVID-19 transmission, which is important to define pandemic and post-pandemic recommendations for health care and self-care focusing this population.

## Author contributions

**Data analysis:** Gilberto Martins Santos, Liliane Pereira Braga, Valter Cordeiro Barbosa Filho, José Carlos Leitão.

**Financing:** The research was funded by the Federal University of Rio Grande do Norte, through the Postgraduate Pro-rectory, and the Postgraduate Program in Public Health. In addition, we thanks the Coordenação de Aperfeiçoamento de Pessoal de Nível Superior - Brasil (CAPES) - Finance Code 001, for the incentive by granting PhD scholarships.

**Metodology:** Gidyenne Christine Bandeira Silva de Medeiros, Kesley Pablo Morais de Azevedo, Victor Hugo de Oliveira Segundo, Ádala Nayana de Sousa Mata, Ana Clara de França Nunes, Isac Davidson Pimenta, Isaac Newton Machado Bezerra.

**Project administration:** Grasiela Piuvezam, Gidyenne Christine Bandeira Silva de Medeiros, Daniel Guillén Martínez.

**Reading and Final Revision of the Text:** All.

**Research in the area of clinical-immunological manifestations:** Márcia Regina Piuvezam.

**Research in the area of treatment of Covid-19 in the infant population:** Helaine Carneiro Capucho and Márcia Regina Piuvezam.

**Research:** All.

**Writing of the scientific paper:** Ana Clara de França Nunes, Gidyenne Christine Bandeira Silva de Medeiros, Kesley Pablo Morais de Azevedo, Victor Hugo de Oliveira Segundo, Grasiela Piuvezam.
